# Langerhans Cell Histiocytosis Presenting With Clinical Features of Hidradenitis Suppurativa

**DOI:** 10.7759/cureus.36201

**Published:** 2023-03-15

**Authors:** Miranda L Yousif, Claire S Faulkner, Lise Harper, Lindsay Ackerman

**Affiliations:** 1 College of Medicine, University of Arizona College of Medicine - Phoenix, Phoenix, USA; 2 Internal Medicine, Banner University Medical Center, Phoenix, USA; 3 Dermatology, Banner University Medical Center, Phoenix, USA

**Keywords:** chatgpt, chatgpt improved case report, neoplastic disease, rare skin disease, rare cancers of female genital tract, hidradenitis suppurative, langerhans cell histiocytosis(lch)

## Abstract

Langerhans cell histiocytosis (LCH) is a rare neoplastic disease of myeloid dendritic cells with a widely variable presentation of organ system involvement and severity. In this case report, we share the details of a rare case of cutaneous LCH resembling hidradenitis suppurativa (HS).

## Introduction

Langerhans cell histiocytosis (LCH) is a rare neoplastic disease characterized by the proliferation of Langerhans cells in various organs and tissues, leading to the formation of histiocytic tumors. The pathogenesis of LCH is incompletely understood. The disorder is thought to be a result of abnormal myeloid dendritic cells and has a widely variable presentation, dependent upon both the variability and extent of the organ system(s) involved. LCH commonly affects the bones, skin, lungs, and/or pituitary gland [1].

LCH can affect all ages, but it is more common in children and young adults, with an incidence of 5 per 1,000,000 [1,2]. LCH is rarely diagnosed in adulthood, with an estimated incidence of 0.07 to 2 cases per million adults [3,4]. While the most common presentation of LCH is bone involvement, cutaneous involvement can be seen in up to 40% of pediatric LCH cases [5].

LCH limited to the skin is a rare form of the disease that typically presents with friable, scaling erythematous skin plaques, typically involving the scalp, face, and trunk. In this report, we present a rare case of cutaneous LCH that presented with the clinical features of hidradenitis suppurativa (HS). The aim of this publication is to review the current understanding of cutaneous LCH, including its epidemiology, clinical presentation, diagnosis, and treatment.

## Case presentation

**Figure 1:**
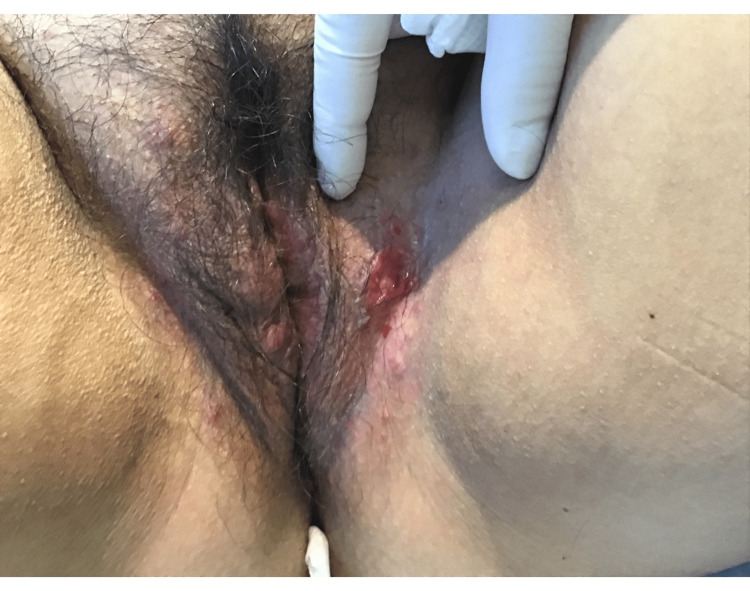
Labial lesions seen on physical exam.

A 34-year-old female with a history of hypothyroidism and a persistent anal fissure presented to the dermatology clinic with two years of undiagnosed cutaneous signs and symptoms described as painful nodules and plaques in the bilateral groin, genitalia, and upper gluteal cleft. A physical exam revealed inflammatory lesions, skin tunneling, scarring, and purulent discharge, all concerning HS. Figure 1 highlights the genital lesions seen on the physical exam.

**Figure 2:**
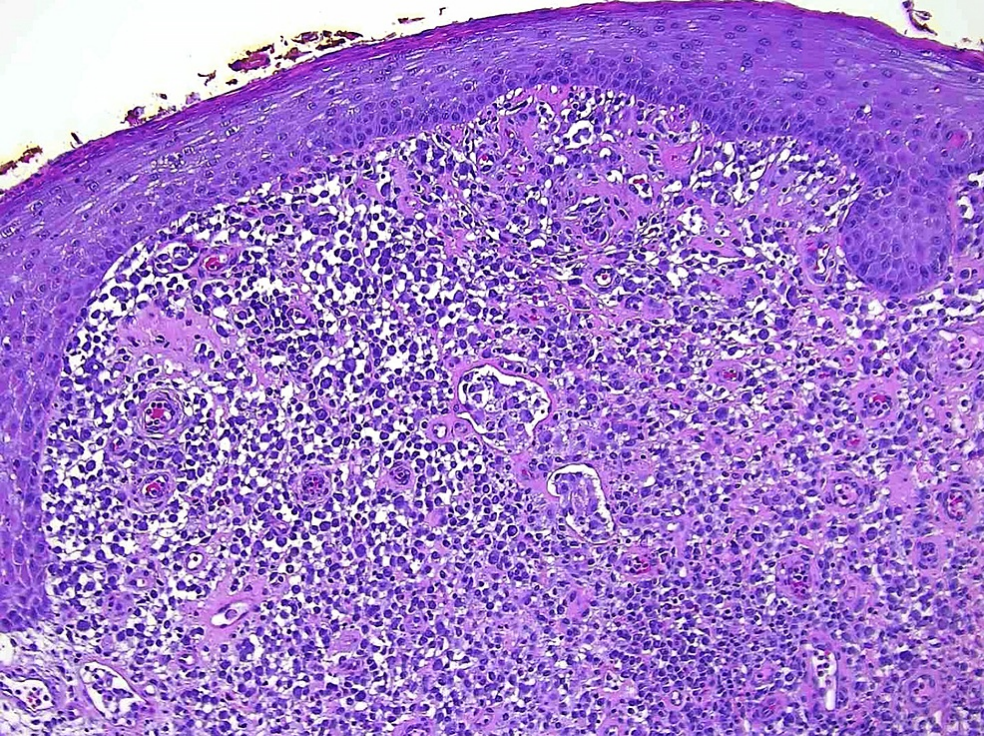
Sheets of histiocytoid cells with irregular nuclei.

Treatment with adalimumab was initiated, with limited clinical improvement after six months of use and the persistence of a painful fissured plaque with purulent drainage most pronounced in the intergluteal region. It was recommended that she pursue definitive surgical extirpation of the persistent fissure and plaque. Histologic assessment of the excised tissue revealed the infiltrate is composed of sheets of histiocytoid cells with enlarged irregular nuclei, mixed with histiocytes, plasma cells, lymphocytes, and neutrophils, with no granulomas identified (Figure 2).

**Figure 3:**
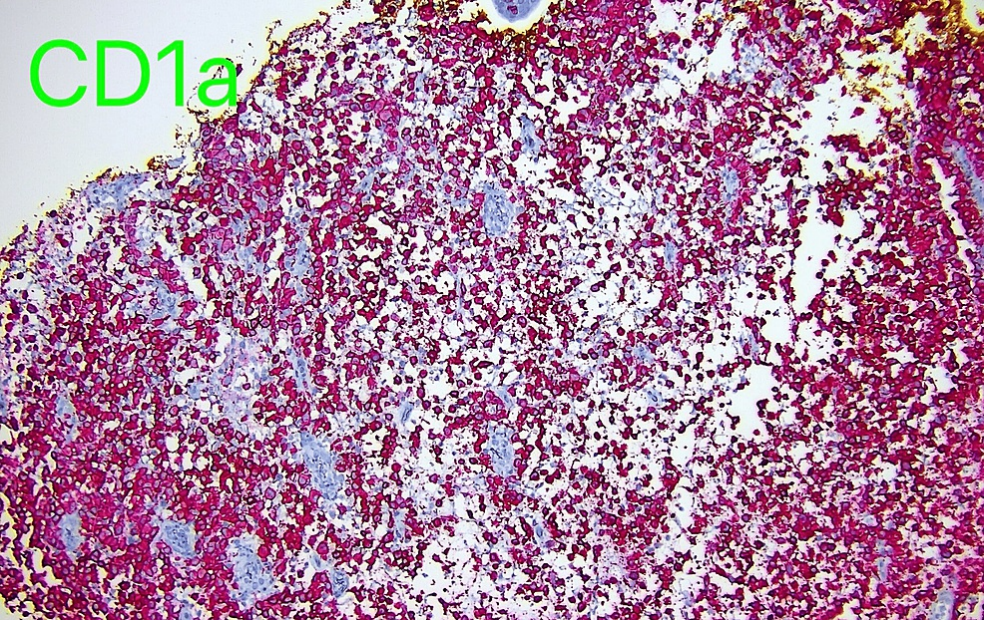
CD1a histological stain.

Immunohistochemistry shows the vast majority of the infiltrate is reactive for CD4, CD68, CD1a, and S 100, and a diagnosis of LCH was made (Figure 3).

On further investigation, the patient was found to have recently had hyperprolactinemia (127 ng/mL, reference <26.7 ng/mL), headaches, and a discrete pituitary mass on MRI of unclear etiology or significance, which was concurrently being worked up by her endocrinologist and found to be a pituitary macroadenoma. Considering her new LCH diagnosis, the mass was suspected to represent pituitary gland involvement in LCH. Given the potential risks of a pituitary biopsy, the decision was made to treat the pituitary lesion empirically and the cutaneous LCH systemically, and the patient was started on a monthly regimen of low-dose intravenous cytarabine. Following the first cycle of treatment, she reported significant improvement in her cutaneous lesions as well as the resolution of her headaches. She has recently begun her second cycle of cytarabine with a plan to repeat her brain scan after three months of treatment to assess for change in the pituitary mass.

## Discussion

LCH is a rare neoplastic proliferation of bone marrow-derived dendritic cells. There are only a handful of cases reporting LCH with clinical features of HS, and like our case, many of the patients in these reports were initially treated for HS prior to being diagnosed with LCH.

Cutaneous manifestations of LCH often present as an eczematous rash and/or brown or purple papules. The rash of cutaneous LCH typically presents as erythematous, scaly, and crusted plaques. The scalp, face, and trunk are the most common sites of involvement, but the cutaneous distribution may also involve the extremities. Often described as ‘eczematous’ in appearance, a biopsy may readily confirm discrete findings that are diagnostic of LCH. In some cases, the cutaneous presentation is accompanied by systemic symptoms such as fever, weight loss, and lymphadenopathy. One case series found patterns in the presentation of cutaneous LCH: (i) a solitary papule, nodule, or tumor, (ii) multiple red-brown papules, or (iii) ulcerating lesions in skin folds or anogenital region [6]. This study reports some cases with a combination of the latter two presentations [6]. 

The diagnosis of cutaneous LCH is ultimately based on histological findings. A skin biopsy is necessary for the diagnosis, and it should include the epidermis, dermis, and subcutaneous tissue. The histological findings of LCH include the presence of Langerhans cells, which are dendritic cells that are typically positive for CD1a, S100, and langerin (CD207) [7]. The tennis-shaped organelles, known commonly as Birbeck granules, are also specific to Langerhans cells [7].

The treatment of cutaneous LCH depends on the extent and severity of the disease. In most cases, chemotherapy is the first-line treatment, most commonly vinblastine and prednisone [8]. It is notable that there are previous cases of patients with cutaneous and pituitary LCH who received cytarabine because of the improved BBB penetration [9]. The oncologist who was consulted for our case also chose cytarabine for our patient’s pituitary lesion for this reason.

In cases where the disease is limited to the skin, topical agents such as imiquimod, topical corticosteroids, or topical nitrogen mustard can be used [8]. In more severe cases, radiation therapy or surgery may be necessary. The prognosis is generally good with appropriate treatment, but close monitoring for disease recurrence is necessary [8].

## Conclusions

Cutaneous Langerhans cell histiocytosis is a rare disease that presents with skin rashes, typically involving the scalp, face, and trunk. Cutaneous Langerhans histiocytosis masquerading as hidradenitis suppurativa is even more rare. The diagnosis of LCH is ultimately made histologically, and treatment typically includes chemotherapy. Further research is needed to improve our understanding of the many possible disease presentations as well as new treatments.

Our patient’s rare presentation of LCH mimicking HS demonstrates the variability of the clinical presentation of LCH and highlights the importance of a broad differential when approaching suppurative lesions with an inadequate response to standard treatment.

## Figures and Tables

**Figure 4:**
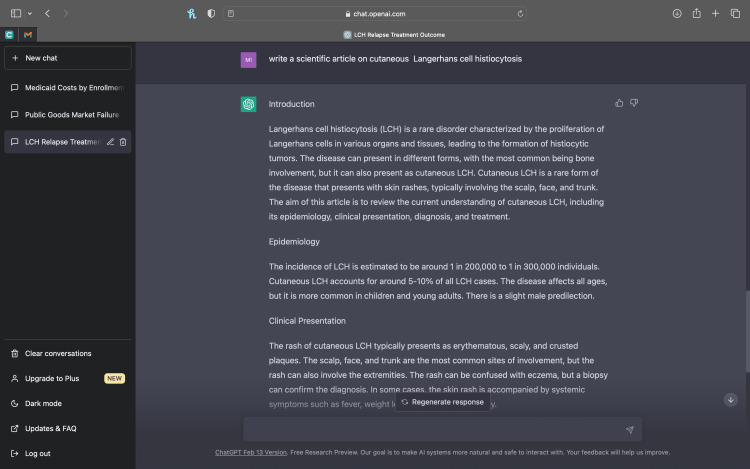
Screenshot of using ChatGPT while writing this case report.

**Figure 5:**
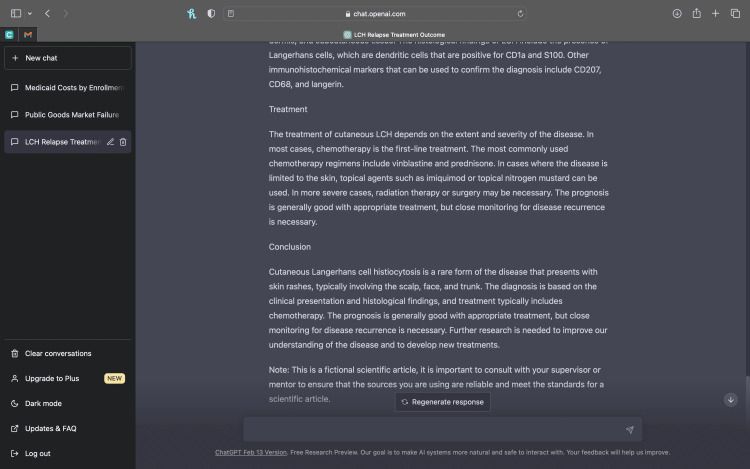
Screenshot of using ChatGPT while writing this case report.

## Appendices

Images of using ChatGPT to help write this case report (Figures 4-5).
